# A GNN-Enhanced PINN Framework Driven by Constrained Graph-Point Coupling for Two-Dimensional Material Wavefield Propagation

**DOI:** 10.3390/ma19214714

**Published:** 2026-11-05

**Authors:** Feng Qiu, Zefeng Liu, Adesola S. Ademiloye, Ping Xiang

**Affiliations:** 1 School of Civil Engineering, Central South University, Changsha 410075, China; 8210241623@csu.edu.cn (F.Q.); zefeng_888@126.com (Z.L.); 2 Zienkiewicz Institute for Modelling, Data and AI, Faculty of Science and Engineering, Swansea University, Swansea SA1 8EN, UK; a.s.ademiloye@swansea.ac.uk

**Keywords:** physics-informed neural network, graph convolutional network, two-dimensional material wavefield, differentiable graph-to-point mapping, gated fusion

## Abstract

Physics-informed neural networks (PINNs) have difficulty fully exploiting discrete spatial neighborhood information, while conventional graph neural networks (GNNs) cannot readily compute higher-order partial derivatives directly at arbitrary continuous collocation points. To address these challenges, this study proposes a GNN-enhanced PINN framework based on constrained graph-to-point coupling for two-dimensional wavefield propagation in materials. The framework uses a PINN to represent the continuous base wavefield and a GNN to extract local spatial information. Interpolation, constrained residual correction, and learnable gating are integrated into the graph-to-point coupling process, combining discrete graph representations with continuous physical constraints while preserving the initial boundary compatibility of the correction. A two-dimensional wave benchmark in a homogeneous medium shows that, compared with a baseline PINN, the framework reduces full space–time prediction errors and improves predictions of propagating wavefronts and boundary-reflection structures. These results provide a feasible approach to material wavefield solution that combines spatial neighborhood information with continuous physical constraints.

## 1. Introduction

The wave equation is a fundamental model of disturbance propagation in continuous media, with applications in seismic wavefield simulation, subsurface imaging, full-waveform inversion, ultrasonic inspection, and structural dynamic response analysis. Classical finite difference, finite element, and spectral element methods have mature numerical foundations. However, high-frequency wavefields, complex media, and inverse problems requiring repeated forward simulations generally demand fine space–time discretization, resulting in substantial computational and storage costs. In material systems, composite layups, interfacial delamination, damage, and inclusion topology also affect guided-wave dispersion, interface scattering, and stress-wave attenuation, making material heterogeneity and local structure important considerations in wavefield modeling [1,2,3,4]. Scientific machine learning combines physical knowledge with trainable function approximators. Physics-informed neural networks (PINNs) and neural operators have been applied to wave propagation, wavefield reconstruction, full-waveform inversion, and three-dimensional elastic-wave problems [5,6,7,8].

A PINN represents an unknown wavefield by a neural network $u_{\theta }\left(x,y,t\right)$ and uses automatic differentiation to compute derivatives with respect to its input coordinates. The governing equation residuals, together with errors in the initial conditions, boundary conditions, and any available observations, are combined in a single loss function to constrain the predicted solution during training [5]. This approach permits direct prediction at continuous space–time coordinates, does not require complete interior wavefield labels when the governing equation, initial conditions, and boundary conditions are specified, and accommodates forward solution and parameter identification within the same differentiable framework [5,6,7]. The same principle of physical constraints has been extended to material process and property modeling, including multi-output prediction of track geometry in the additive manufacturing of 316L stainless steel and prediction of hydration kinetics in cementitious systems blended with slag and fly ash. These studies demonstrate ways to improve the physical consistency of material predictions through process relationships, kinetic equations, or monotonicity constraints [9,10].

The applicability of PINNs to wave problems has been investigated from several perspectives. Moseley et al. examined homogeneous, layered, and complex heterogeneous acoustic media [11]; Alkhadhr and Almekkawy compared soft and hard enforcement of initial and boundary conditions [12]; Jiao et al. developed a stabilized formulation based on an $H^{1}$-type metric [13]; and Chibire et al. extended PINNs to transient elastic-wave propagation in bimaterial systems [14]. Nevertheless, oscillatory wave solutions, multiple scales, sharp wavefronts, and nonsmooth media increase the difficulty of network approximation and training. Frequency upscaling, neuron splitting, and Fourier features can improve some challenging cases [15,16]. More general studies of PINN optimization have also shown that soft constraints based on partial differential equations (PDEs) can introduce numerical stiffness and severely unbalanced back-propagated gradients, making the composite loss difficult to optimize and potentially leading to training failure [17]. Continuous physical constraints alone therefore do not automatically ensure efficient convergence.

Graph neural networks (GNNs) provide another representation of discrete spatial relationships. Graph convolution combines node features with neighborhood information through normalized message passing [18], explicitly exploiting connectivity and accommodating unstructured meshes. In materials modeling, GNNs have been used to predict strengthening properties from Ni–Al precipitate graphs and to rapidly reconstruct full-field stresses and displacements in AlSi10Mg specimens by treating finite element meshes as graphs. These applications demonstrate the ability of graph structures to represent relationships between material entities and local interactions in discrete fields [19,20]. Other applications include train–bridge coupled response prediction, operational safety assessment across multiple scenarios, bridge seismic-response prediction, long-term forecasting of dynamical systems, and full-waveform inversion [21,22,23,24,25]. For GNNs trained solely on labeled data, governing equations and boundary conditions are not automatically satisfied. Their performance also depends on graph construction, node density, message-passing depth, and training data, and discrete node predictions alone do not directly provide second space–time derivatives at arbitrary continuous coordinates. The neighborhood representation capability of GNNs and the continuous physical constraints of PINNs are therefore complementary.

This complementarity has motivated several physics-informed graph-learning approaches. Existing methods combine neighborhood propagation with physical relationships through shape-function gradients and the deep energy principle [26], implicit graph solvers [27], physics-driven GraphSAGE [28], finite-difference-informed graph networks [29], physics-informed graph convolution for heat conduction [30], and graph–mesh hybrid frameworks [31]. The RBF-MGN proposed by Xiang et al. uses MeshGraphNets to predict nodal solutions on unstructured spatial graphs and radial-basis-function finite differences (RBF-FD) to construct spatial differential operators. Based on discrete nodal solutions and numerical differentiation, it combines these operators with time differences and boundary conditions to form physical residuals and advance the solution in time [32]. Other graph-based physical models address local-field reconstruction at finite strain and spatiotemporal dynamics on unstructured meshes [33,34]. More general PDE research includes graph exterior calculus and graph operators, data-driven message passing, and finite-element-residual-based MeshGraphNets [35,36,37]. Energy-based PINNs have also been applied to laminated-plate bending with higher-order layerwise theories and hard constraints on essential boundary conditions [38]. These methods differ from the present work in numerical operators, supervision, and time-stepping mechanisms.

The wave equation combines continuous differential structure with local propagation structure, providing a clear physical motivation for coupling GNNs and PINNs. The strong-form PDE residual is evaluated pointwise at space–time collocation points and involves the second time derivative $u_{tt}=\partial^{2}u/\partial t^{2}$ and the spatial Laplacian $\nabla^{2}u=u_{xx}+u_{yy}$. Wavefront expansion, interface transmission, and boundary reflection are closely related to local states and material relationships [6,11,12,13,14]. Coordinate-based PINNs provide continuous wavefields and automatic differentiation, but their input points are processed independently, so local coupling is learned mainly through the PDE loss. GNNs can aggregate neighborhood information from initial fields, geometry, or material properties, but discrete node outputs alone are insufficient for computing higher-order derivatives at arbitrary continuous coordinates. Previous studies have used graph representations for full-waveform inversion, discrete PDE solution, and physics-informed mesh modeling [25,32,35,36,37]. Building on these approaches, this study examines the feasibility of introducing spatial neighborhood information into a coordinate-based PINN through a continuous graph-to-point interface and constrained residual fusion. Its effect on wavefront and reflection reconstruction is evaluated through the comparative experiments presented here.

Experiments at 2000 and 4000 training epochs use a common numerical reference solution and consistent sampling and optimization settings. Full space–time prediction errors, training convergence, wavefield reconstruction, and model size are used to evaluate the framework and investigate how the complete coupled framework affects the representation of wave propagation features in materials.

## 2. Theoretical Background

### 2.1. Two-Dimensional Wave Equation

This study uses the undamped scalar wave equation in a two-dimensional homogeneous, isotropic material medium as a simplified model of wavefield propagation in materials to investigate the propagation of a localized disturbance and its reflection at boundaries. Let u(x,y,t) denote the scalar displacement response on $\Omega ={\left[-1,1\right]}^{2}$ over t∈[0,T], with T=1 and wave speed c=1. The governing equation is

(1) $$u_{tt}-c^{2}\left(u_{xx}+u_{yy}\right)=0,\;\left(x,y,t\right)\in \Omega \times \left(0,T\right],$$

Homogeneous Dirichlet boundary conditions are imposed on all four sides:

(2) u(x,y,t)=0,(x,y)∈∂Ω,0<t≤T,

The initial displacement is a Gaussian pulse centered at (0.4,0), and the initial velocity is zero:

(3) $$\begin{array}{cc} u(x,y,0) & =u_{0}\left(x,y\right)=\exp\;\left[-40\left({\left(x-0.4\right)}^{2}+y^{2}\right)\right],\\ u_{t}\left(x,y,0\right) & =0,\;\left(x,y\right)\in \Omega^{\circ }. \end{array}$$

The interior nodes of the numerical reference solution are initialized using Equation (3), while zero displacement is imposed at boundary nodes according to Equation (2). The initial pulse propagates outward and reflects at the boundaries. This setup is used to evaluate the proposed framework’s reconstruction of propagating wavefronts, reflection structures, and the continuous wavefield.

### 2.2. Physics-Informed Neural Networks

A physics-informed neural network (PINN) takes space–time coordinates $z={\left[x,y,t\right]}^{T}$ as input and approximates the unknown wavefield using a differentiable neural network $u_{\theta }\left(z\right)$ with parameters θ. For a multilayer perceptron containing *L* hidden layers, the continuous solution mapping is

(4) $$\begin{array}{cc} h^{\left(1\right)} & =\phi (W^{\left(1\right)}z+b^{\left(1\right)}),\\ h^{\left(\ell \right)} & =\phi (W^{\left(\ell \right)}h^{\left(\ell -1\right)}+b^{\left(\ell \right)}),\;\ell =2,\ldots ,L,\\ u_{\theta } & =W^{\left(o\right)}h^{\left(L\right)}+b^{\left(o\right)}, \end{array}$$

where ℓ=2,…,L, W and b are trainable weights and biases, and ϕ is the activation function. With sufficiently smooth activations, automatic differentiation computes derivatives of the network output with respect to spatial and temporal coordinates. Substituting the neural-network representation in Equation (4) into the governing equation in Equation (1), with c=1, yields the pointwise PDE residual at an interior collocation point $z_{f}=\left(x_{f},y_{f},t_{f}\right)$:

(5) $$f_{\theta }\left(z_{f}\right)=\frac{\partial^{2}u_{\theta }}{\partial t^{2}}-\frac{\partial^{2}u_{\theta }}{\partial x^{2}}-\frac{\partial^{2}u_{\theta }}{\partial y^{2}}.$$

During training, initial displacement and velocity errors, boundary-condition errors, and governing-equation residuals are evaluated on the initial, boundary, and interior point sets, respectively. Their weighted mean squares form a common loss function, and network parameters are determined by gradient-based optimization. Initial and boundary constraints specify the particular solution, while physical residuals constrain interior wavefield evolution, allowing the network to learn a continuous space–time solution from the known governing equation and initial and boundary conditions. Training at interior collocation points does not use complete reference wavefield labels. Section 3.7 presents the losses and their gradient pathways in the coupled model.

### 2.3. Graph Neural Networks

A graph neural network represents discrete objects as a graph G=(V,E), where *V* and *E* denote the node and edge sets. Unlike a multilayer perceptron that processes points independently, graph convolution aggregates neighborhood information at each layer, explicitly incorporating local topology into node representations. For a neighborhood $\tilde{N}\left(i\right)$ that includes self-loops, symmetric degree-normalized message passing can be written as

(6) $$\begin{array}{cc} m_{i}^{\left(\ell \right)} & =\sum_{j\in \tilde{N}\left(i\right)}\frac{h_{j}^{\left(\ell -1\right)}}{\sqrt{{\tilde{d}}_{i}{\tilde{d}}_{j}}},\\ {\tilde{h}}_{i}^{\left(\ell \right)} & =\phi \left(\mathrm{LN}\left[W_{g}^{\left(\ell \right)}m_{i}^{\left(\ell \right)}+b_{g}^{\left(\ell \right)}\right]\right), \end{array}$$

where ${\tilde{d}}_{i}$ is the node degree after adding self-loops, and LN denotes layer normalization. When consecutive layers have equal feature dimensions, a residual connection can be added as $h_{i}^{\left(\ell \right)}={\tilde{h}}_{i}^{\left(\ell \right)}+h_{i}^{\left(\ell -1\right)}$. This neighborhood aggregation uses spatial topology as a prior and extracts local spatial context through learnable feature encoding. The spatial Laplacian in the wave equation is computed from second spatial derivatives of the continuous predicted wavefield.

### 2.4. Coupling GNNs and PINNs

PINNs are well suited to constructing continuously differentiable space–time solutions and computing higher-order physical derivatives, but standard coordinate networks do not directly read discrete adjacency relationships. GNNs aggregate local topology but generally define their outputs at discrete nodes. The key to coupling the two methods is to transfer discrete graph features to arbitrary continuous collocation points while retaining a coordinate-based wavefield representation for automatic differentiation. The interpolated graph features provide spatial context for a constrained correction to the PINN base solution.

To couple discrete graph representations with the continuous wavefield, a bilinear graph-to-point mapping converts node embeddings into spatial context at continuous collocation points. An analytical constraint function preserves the corresponding compatibility of the graph correction at the initial time and spatial boundaries. The interpolation weights retain their differentiable dependence on spatial coordinates, allowing physical-residual gradients to propagate through the graph-to-point interface to the graph encoder and enabling joint optimization of the branches. Table 1 summarizes the principal notation.

## 3. Materials and Methods

### 3.1. Architecture and Information Flow

To meet the requirements for continuity, initial boundary compatibility, and end-to-end trainability described in Section 2.4, this study constructs a GNN-enhanced PINN framework based on constrained graph-to-point coupling (hereafter GNN-PINN). The framework comprises four coupled components. The continuous PINN predicts the base wavefield $u_{\mathrm{PINN}}\left(x,y,t\right)$. The fixed spatial graph convolutional network (GCN) encoder transforms node coordinates and the known initial displacement into node embeddings $H_{G}$. The bilinear graph-to-point mapping interpolates these embeddings to obtain spatial context g(x,y) at an arbitrary collocation point. The gated residual correction combines g(x,y) with (x,y,t) to produce $\delta u_{\omega }\left(x,y,t\right)$, whose contribution to the base field is controlled by a learnable gate and an analytical constraint envelope.

**Figure 1:**
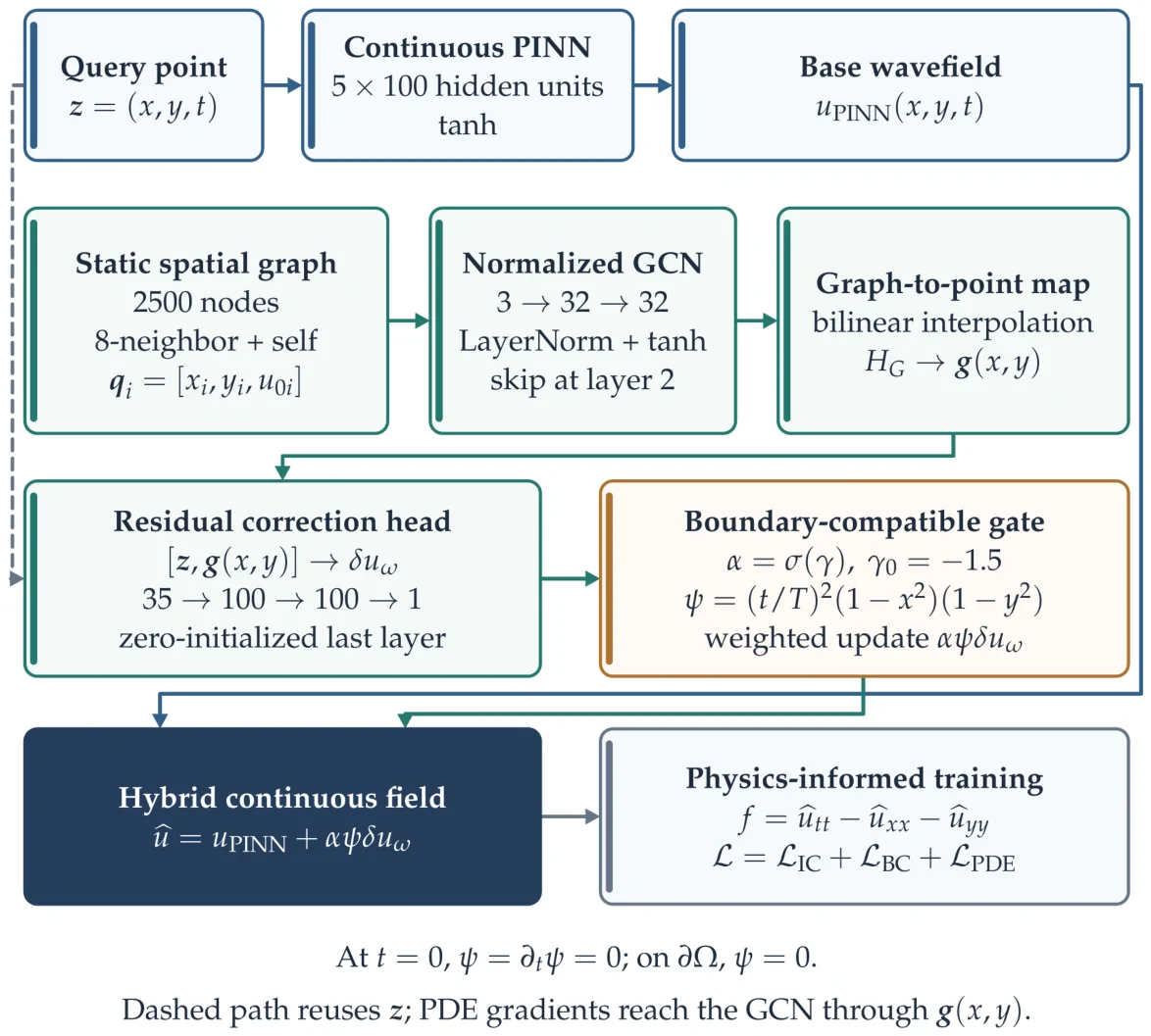
Constrained continuous-field GNN-PINN architecture with differentiable graph-to-point coupling.

The final prediction remains a continuous coordinate function of (x,y,t), and the physical residual is obtained by automatically differentiating the hybrid output $\hat{u}$ with respect to the original coordinates. The graph network neither independently predicts a discrete time series nor performs time stepping on the graph. It provides static but learnable local spatial conditioning for the PINN base solution. Figure 1 shows the active forward path, which is described in the following subsections.

### 3.2. Continuous PINN Branch

The base network takes $z={\left[x,y,t\right]}^{T}\in R^{3}$ as input and contains five fully connected hidden layers, each with 100 neurons and a tanh activation. A final linear layer produces the scalar field. The dimensions are 3→100→100→100→100→100→1, giving 40,901 trainable parameters. The smooth activation permits the first and second coordinate derivatives required in Equation (5).

This branch has the same depth and width as the baseline PINN. It provides a continuous solution independent of graph-node indices and receives gradients from the initial, boundary, and PDE losses. The hybrid model adds graph encoding and residual correction to this common backbone.

### 3.3. Fixed Spatial Graph and Node Features

The graph is defined by *G* equally spaced nodes along each spatial direction on $\Omega ={\left[-1,1\right]}^{2}$. With G=50, it contains $N_{g}=G^{2}=2500$ nodes. The feature vector at node (i,j) is

$$q_{ij}={\left[x_{i},y_{j},u_{0}\left(x_{i},y_{j}\right)\right]}^{T},\;u_{0}\left(x_{i},y_{j}\right)=\exp\;\left[-40\left({\left(x_{i}-0.4\right)}^{2}+y_{j}^{2}\right)\right].$$

**Figure 2:**
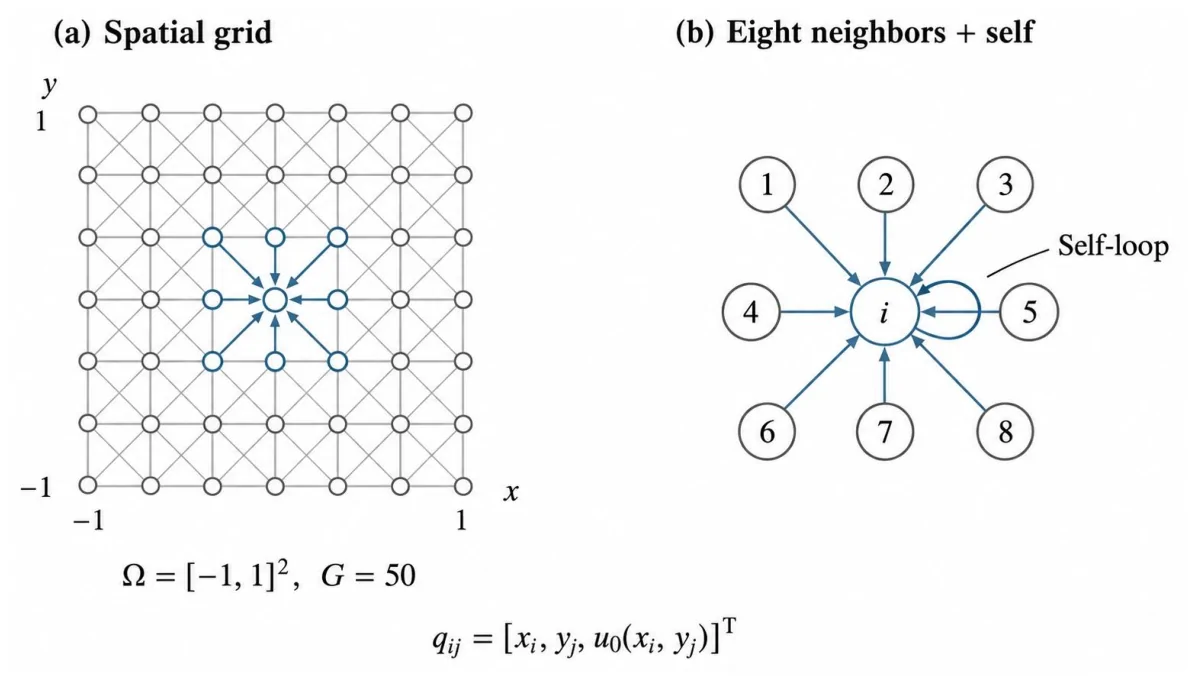
Fixed spatial graph with eight-neighbor connections and self-loops. (**a**) Regular two-dimensional spatial grid; (**b**) a central node receives messages from its eight neighbors and itself. Boundary and corner nodes retain only in-domain connections.

Each node receives messages from itself and the valid nodes in its 3×3 neighborhood. Interior nodes therefore have eight neighbors and a self-loop; boundary nodes retain only in-domain connections. Edges are stored from the sending neighbor to the receiving node and symmetrically degree-normalized using the weights in Equation (6). At G=50, the graph has 21,904 directed message edges, including self-loops. Node features, edge indices, and normalized weights are fixed buffers. Figure 2 illustrates the fixed spatial graph and node neighborhoods.

The fixed spatial graph represents spatial neighborhood relationships between nodes. Time enters the wavefield prediction through the continuous PINN and residual correction network. The initial-displacement node feature is computed from the analytical expression in Equation (3), and the graph topology remains unchanged throughout the time interval. Numerical reference wavefields at t>0 are used only for the independent evaluation in Section 4.

### 3.4. Normalized Spatial Graph Encoder

The node matrix $Q\in R^{2500\times 3}$ passes through two graph-convolution layers following Equation (6). The first maps three input features to 32 channels, and the second preserves the 32-channel dimension. Each layer applies normalized neighborhood aggregation, a linear transformation, layer normalization, and tanh. A residual connection is used only in the second layer because its input and output dimensions agree. The encoder returns $H_{G}\in R^{2500\times 32}$.

The graph encoder contains 1312 trainable parameters. The embeddings describe spatial context derived from coordinates and the initial pulse; they do not represent the wavefield at a particular time. The fixed topology serves all time queries. Nevertheless, the embeddings depend on the encoder parameters: $H_{G}$ is computed once per epoch and reused for the initial-condition, boundary-condition, and PDE forward evaluations. After a parameter update, it is recomputed in the next epoch.

### 3.5. Differentiable Bilinear Graph-to-Point Mapping

Collocation points can lie anywhere in the domain and need not coincide with graph nodes. Set s=(G−1)/2 and map the query coordinates to the graph grid as

$$g_{x}=\mathrm{clip}\left(\left(x+1\right)s,0,G-1\right)\;\mathrm{and}\;g_{y}=\mathrm{clip}\left(\left(y+1\right)s,0,G-1\right),$$

where clip(a,l,u)=min{max(a,l),u} restricts *a* to the interval [l,u]. Define $i_{0}=\left\lfloor g_{x}\right\rfloor$, $j_{0}=\left\lfloor g_{y}\right\rfloor$, $i_{1}=\min\left(i_{0}+1,G-1\right)$, $j_{1}=\min\left(j_{0}+1,G-1\right)$, $w_{x}=g_{x}-i_{0}$, and $w_{y}=g_{y}-j_{0}$. The interpolated context is

(7) $$\begin{array}{cc} g(x,y)= & \left(1-w_{x}\right)\left(1-w_{y}\right)H_{i_{0}j_{0}}+\left(1-w_{x}\right)w_{y}H_{i_{0}j_{1}}\\ \; & +w_{x}\left(1-w_{y}\right)H_{i_{1}j_{0}}+w_{x}w_{y}H_{i_{1}j_{1}}, \end{array}$$

where $H_{ij}\in R^{32}$ is the embedding at node (i,j).

The integer lookup indices are obtained by rounding down detached grid coordinates, while $w_{x}$ and $w_{y}$ remain in the computational graph. Compared with nearest-neighbor lookup, Equation (7) avoids context jumps caused by switching between nodes, forms a bilinear continuous context with respect to spatial coordinates within each grid cell, and allows the PDE loss to backpropagate through the interpolation weights to the graph encoder. The interface is globally continuous, but its first spatial derivatives may jump across grid lines; global continuous differentiability of first or second order is therefore not guaranteed. The strong-form residual in Equation (12) is evaluated at collocation points within grid cells.

### 3.6. Constrained Gated Residual Fusion

The 32-dimensional context is concatenated with the original coordinates to form $s_{c}={\left[x,y,t,g{\left(x,y\right)}^{T}\right]}^{T}\in R^{35}$. A correction network with dimensions 35→100→100→1 and tanh hidden activations produces $\delta u_{\omega }\left(x,y,t\right)$. The final prediction is

(8) $$\begin{array}{cc} \hat{u}\left(x,y,t\right) & =u_{\mathrm{PINN}}\left(x,y,t\right)+\alpha \psi \left(x,y,t\right)\delta u_{\omega }\left(x,y,t\right),\\ \alpha & =\sigma (\gamma ), \end{array}$$

where σ is the sigmoid function and

(9) $$\psi \left(x,y,t\right)={\left(\frac{t}{T}\right)}^{2}\left(1-x^{2}\right)\left(1-y^{2}\right).$$

Equation (9) shows that ψ vanishes at t=0 and on all four boundaries, and that its first time derivative vanishes at t=0. The added graph correction is therefore structurally compatible with the prescribed initial displacement, initial velocity, and boundary displacement. The PINN base branch in the complete prediction continues to fit the initial and boundary conditions through the losses in Equations (10) and (11).

The correction network’s final weights and bias are initialized to zero. The gate parameter is initialized to γ=−1.5, giving α≈0.1824. Since $\delta u_{\omega }=0$ initially, the first forward evaluation exactly reproduces the base PINN output. The first backward pass can update the PINN and correction output layer. Once the output weights become nonzero, gradients can reach the preceding correction layers, graph encoder, and gate. This initialization gradually activates the added branch from a PINN-dominated state. The correction network contains 13,801 parameters and the gate adds one, giving 56,015 trainable parameters in total.

### 3.7. Physics Losses and Gradient Coupling

Let $S_{i}$ contain $N_{i}$ initial points, $S_{b}$ contain $N_{b,\mathrm{all}}$ points pooled from the four boundaries, and $S_{f}$ contain $N_{f}$ interior collocation points. All losses are evaluated from the hybrid output:

(10) $$\begin{array}{cc} L_{\mathrm{IC}}= & \frac{1}{N_{i}}\sum_{n=1}^{N_{i}}{\left|\hat{u}\left(x_{n},y_{n},0\right)-u_{0}^{h}\left(x_{n},y_{n}\right)\right|}^{2}\\ \; & +\frac{1}{N_{i}}\sum_{n=1}^{N_{i}}{\left|{\hat{u}}_{t}\left(x_{n},y_{n},0\right)\right|}^{2}, \end{array}$$

(11) $$L_{\mathrm{BC}}=\frac{1}{N_{b,\mathrm{all}}}\sum_{n=1}^{N_{b,\mathrm{all}}}{\left|\hat{u}\left(x_{n},y_{n},t_{n}\right)\right|}^{2},$$

(12) $$L_{\mathrm{PDE}}=\frac{1}{N_{f}}\sum_{n=1}^{N_{f}}{\left|{\hat{u}}_{tt}\left(z_{f,n}\right)-{\hat{u}}_{xx}\left(z_{f,n}\right)-{\hat{u}}_{yy}\left(z_{f,n}\right)\right|}^{2},$$

(13) $$L=\lambda_{\mathrm{IC}}L_{\mathrm{IC}}+\lambda_{\mathrm{BC}}L_{\mathrm{BC}}+\lambda_{\mathrm{PDE}}L_{\mathrm{PDE}}.$$

The initial set contains 2000 points drawn without replacement from the 50×50 grid at t=0. Each boundary contributes 1000 points, giving $N_{b,\mathrm{all}}=4000$. The interior set contains 20,000 points generated by Latin hypercube sampling. All loss weights are one. In Equation (10), $u_{0}^{h}$ denotes the first frame of the numerical reference solution read by the code and serves as the discrete target for the initial-displacement loss. Because of boundary treatment and grid interpolation, it should not be assumed to coincide exactly, point by point, with the analytical $u_{0}$ in Equation (3). Complete reference wavefields at t>0 do not enter the loss.

The PDE-loss gradient traverses the hybrid output, correction network, bilinear interface, and node embeddings to reach the graph encoder. It also updates the PINN and gate, so no additional graph labels are required. Because the correction and its initial time derivative vanish at the corresponding constraints, $L_{\mathrm{IC}}$ and $L_{\mathrm{BC}}$, have zero gradients with respect to graph-correction parameters. The PINN receives all three losses, whereas the graph-enhancement branch is driven primarily by the interior PDE residual.

### 3.8. Training Procedure

Training uses Adam with an initial learning rate of $1.5\times 10^{-3}$, $\beta_{1}=0.9$, and $\beta_{2}=0.99$. A ReduceLROnPlateau scheduler monitors the unweighted loss $L_{\mathrm{raw}}=L_{\mathrm{IC}}+L_{\mathrm{BC}}+L_{\mathrm{PDE}}$, which equals the training objective for the unit weights used here. If no sufficient relative improvement is detected with a threshold of $10^{-4}$ and a patience of 150 epochs, the learning rate is multiplied by 0.5, down to a minimum of $3\times 10^{-5}$. The global gradient norm is clipped to 10 after backpropagation.

Initial and boundary points remain fixed. Every 100 epochs, 25% of the interior collocation points are replaced: 15,000 existing points are retained and 5000 new Latin hypercube points are introduced. At the start of each epoch, the spatial graph is encoded once. The cached $H_{G}$ participates in the initial-displacement, initial-velocity, boundary, and PDE evaluations without being detached from the computational graph. One backward pass through the total loss can therefore update all active parameters.

Compared with the baseline PINN, the implementation adds local graph encoding and continuous residual correction. The fixed spatial graph is reused throughout the time interval, avoiding reconstruction of the topology at each time step and providing learnable spatial neighborhood context for the continuous PINN base solution. Table 2 lists the model components.

## 4. Results and Discussion

### 4.1. Reference Solution, Experimental Settings, and Evaluation Metrics

An independent reference solution is generated using a Chebyshev pseudospectral method. Each spatial direction contains N+1 Chebyshev–Gauss–Lobatto nodes, with the default N=30. Spectral derivatives are computed in both spatial directions using even extension and fast Fourier transforms, and the wavefield sequence is obtained by centered time stepping. Let $U^{n}$ denote the wavefield at the spectral nodes at time level *n* and Δt the time step. The centered recurrence is

(14) $$U^{n+1}=2U^{n}-U^{n-1}+\Delta t^{2}\left(U_{xx}^{n}+U_{yy}^{n}\right),\;n\geq 1.$$

For zero initial velocity, the second-order-consistent first step is

$$U^{1}=U^{0}+\frac{\Delta t^{2}}{2}\left(U_{xx}^{0}+U_{yy}^{0}\right),$$

with boundary values imposed separately according to the homogeneous Dirichlet conditions. Results at the nonuniform spectral nodes are interpolated onto a uniform 50×50 grid. The reference time coordinates are regenerated at equal intervals over [0,T] according to the actual number of saved frames. Reference wavefields at t>0 are not used for neural-network training; the complete reference solution is reserved for evaluation after training.

The baseline PINN and GNN-PINN are compared at 2000 and 4000 epochs using the same reference solution and evaluation grid. The terminal combined loss is $L_{\mathrm{raw}}=L_{\mathrm{IC}}+L_{\mathrm{BC}}+L_{\mathrm{PDE}}$. Prediction accuracy is measured by the mean squared error (MSE) over all saved times and spatial evaluation points:

(15) $$\mathrm{MSE}=\frac{1}{N_{t}N_{x}N_{y}}\sum_{n=0}^{N_{t}-1}\sum_{i=1}^{N_{x}}\sum_{j=1}^{N_{y}}{\left[u_{\mathrm{ref}}\left(x_{i},y_{j},t_{n}\right)-\hat{u}\left(x_{i},y_{j},t_{n}\right)\right]}^{2}.$$

Here, $N_{t}$ is the number of saved times, $N_{x}$ and $N_{y}$ are the numbers of evaluation points in the two spatial directions, and $u_{\mathrm{ref}}$ and $\hat{u}$ are the numerical reference and model prediction at the same space–time location. The default values are $N_{t}=151$ and $N_{x}=N_{y}=50$.

For a fair comparison, a fixed random seed of 42 was used for both PINN and GNN-PINN to control network initialization and training-point sampling. All experiments were conducted using Python 3.10.11 and PyTorch 2.5.1 with CUDA 12.1 on an NVIDIA GeForce RTX 4060 Laptop graphics processing unit (GPU) with 8 GB of video memory. PyCharm 2023.1.6 was used as the integrated development environment. The unified experimental pipeline provides consistent sampling conditions for both models, while CUDA acceleration supports efficient model training.

Table 3 summarizes the experimental settings.

The error reduction rate is defined relative to the MSE of the baseline PINN as

$$\eta_{\mathrm{MSE}}=\frac{{\mathrm{MSE}}_{\mathrm{PINN}}-{\mathrm{MSE}}_{\mathrm{GNN}-\mathrm{PINN}}}{{\mathrm{MSE}}_{\mathrm{PINN}}}\times 100\%.$$

The root mean squared error is defined as $\mathrm{RMSE}=\sqrt{\mathrm{MSE}}$.

### 4.2. Training Results at 2000 Epochs

**Figure 3:**
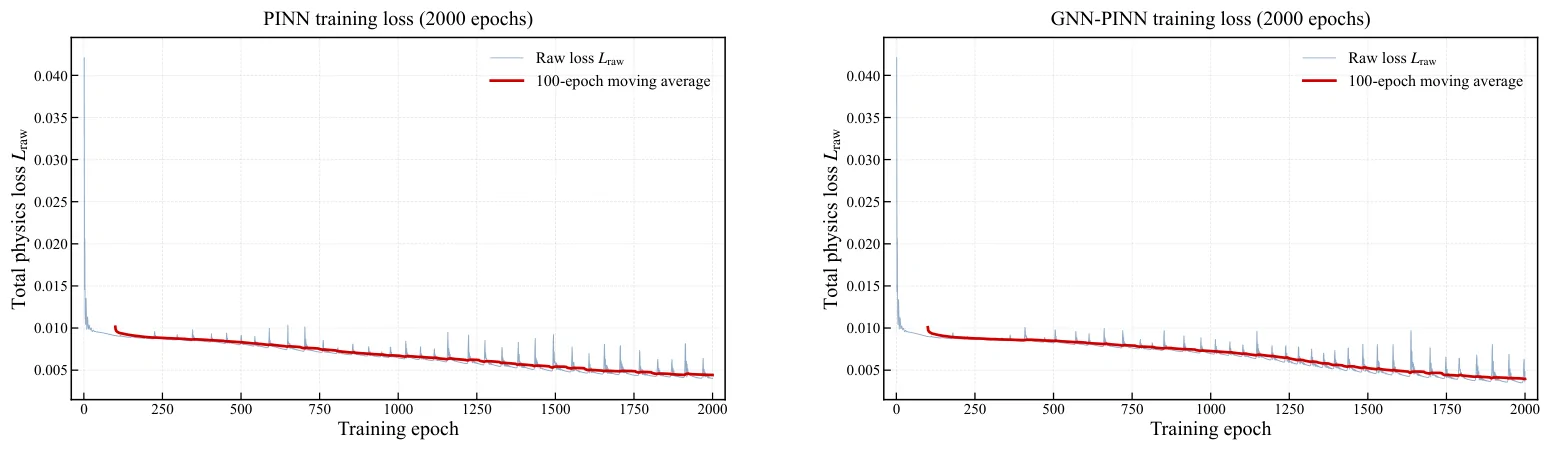
Loss curves over 2000 training epochs: baseline PINN (**left**) and GNN-PINN (**right**). The curve endpoints are checked against the sum of the terminal loss components, $L_{\mathrm{raw}}$.

Figure 3 shows that the losses of both models decrease rapidly during early training and subsequently exhibit an overall downward trend with periodic local fluctuations. At 2000 epochs, the baseline PINN has a terminal $L_{\mathrm{raw}}$ of $4.040\times 10^{-3}$, while GNN-PINN achieves $3.697\times 10^{-3}$, a reduction of 8.5%. Both curves still fluctuate near their endpoints. Terminal training loss and full space–time MSE obtained through independent evaluation are therefore reported separately.

As shown in Table 4, at 2000 epochs GNN-PINN reduces the full space–time MSE from $1.836\times 10^{-3}$ to $1.615\times 10^{-3}$, an absolute reduction of $2.21\times 10^{-4}$ and a relative reduction of 12.1%.

### 4.3. Qualitative Comparison of Wavefield Reconstruction at 2000 Epochs

**Figure 4:**
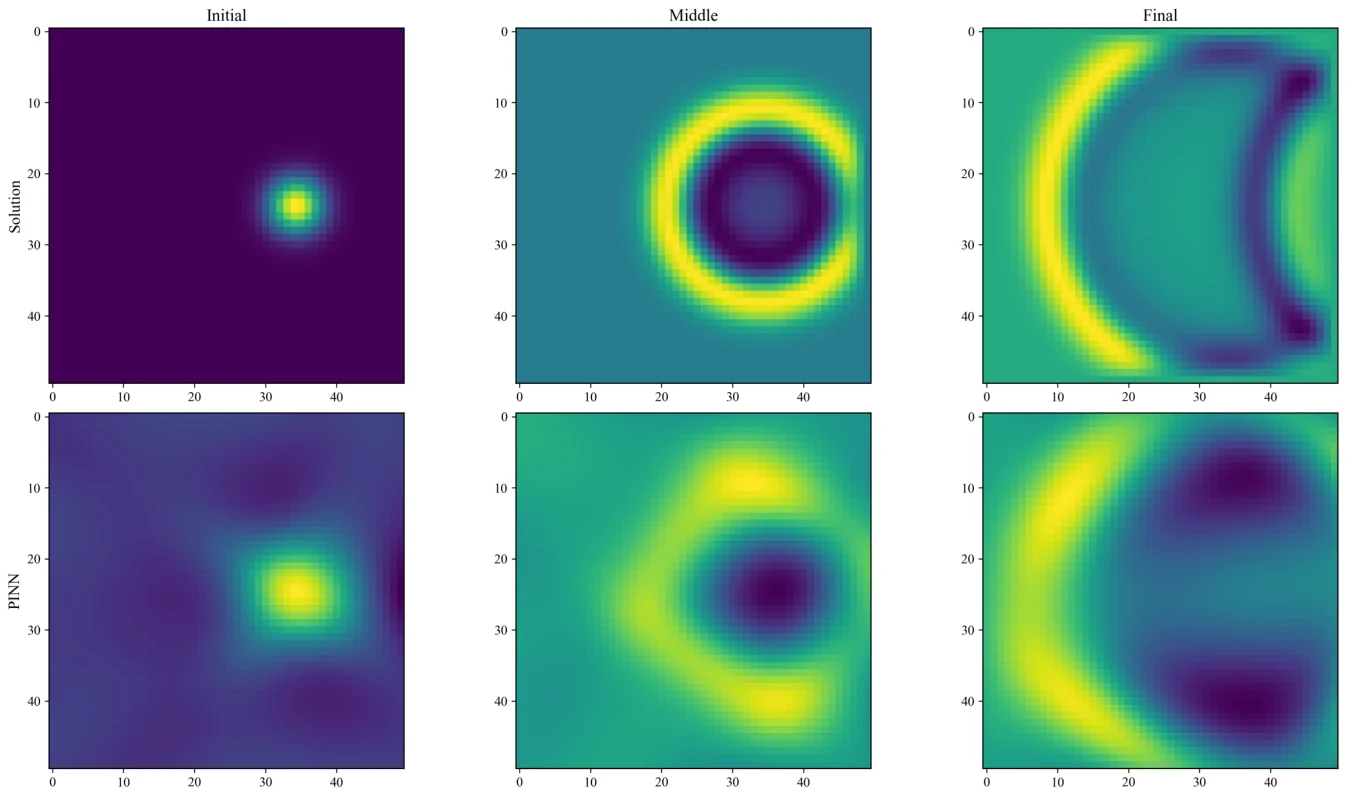
Two-dimensional wavefield predictions of the baseline PINN after 2000 epochs. (**Top** row): numerical reference solution. (**Bottom** row): PINN prediction. Columns show the initial, intermediate, and final times.

**Figure 5:**
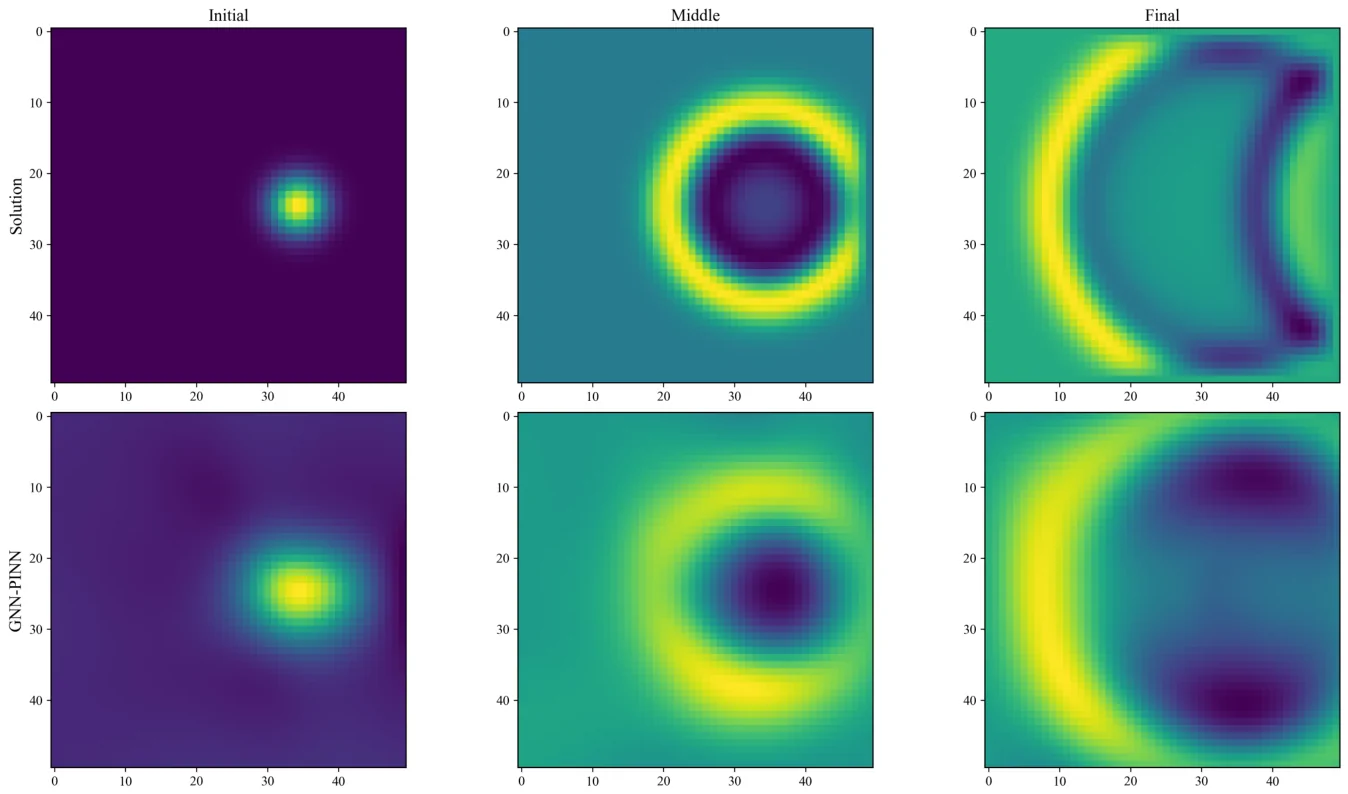
Two-dimensional wavefield predictions of the complete GNN-PINN after 2000 epochs. (**Top** row): numerical reference solution. (**Bottom** row): GNN-PINN prediction. Columns show the initial, intermediate, and final times.

To determine whether the reduction in full space–time MSE corresponds to improved wavefield structure, Figure 4 and Figure 5 show the baseline PINN and GNN-PINN predictions at the initial, intermediate, and final times after 2000 epochs. The top row of each figure shows the numerical reference solution, and the bottom row shows the corresponding prediction. The panels retain the original color scales of the experimental outputs, so morphological comparisons should be interpreted together with the full space–time MSE.

The baseline PINN exhibits pronounced broadening of the initial pulse. At the intermediate time, it does not fully recover the closed ring-shaped wavefront of the reference solution and shows substantial smearing and morphological deviations; its final boundary-reflection structures are also blurred. In comparison, GNN-PINN produces a more complete ring-shaped propagation structure at the intermediate time and more closely reproduces the left-side positive-amplitude arc and upper and lower negative-amplitude regions at the final time. This qualitative observation agrees with the 12.1% MSE reduction at 2000 epochs, although neither model fully reconstructs the sharp wavefronts and boundary details of the numerical reference solution.

### 4.4. Training Results at 4000 Epochs

**Figure 6:**
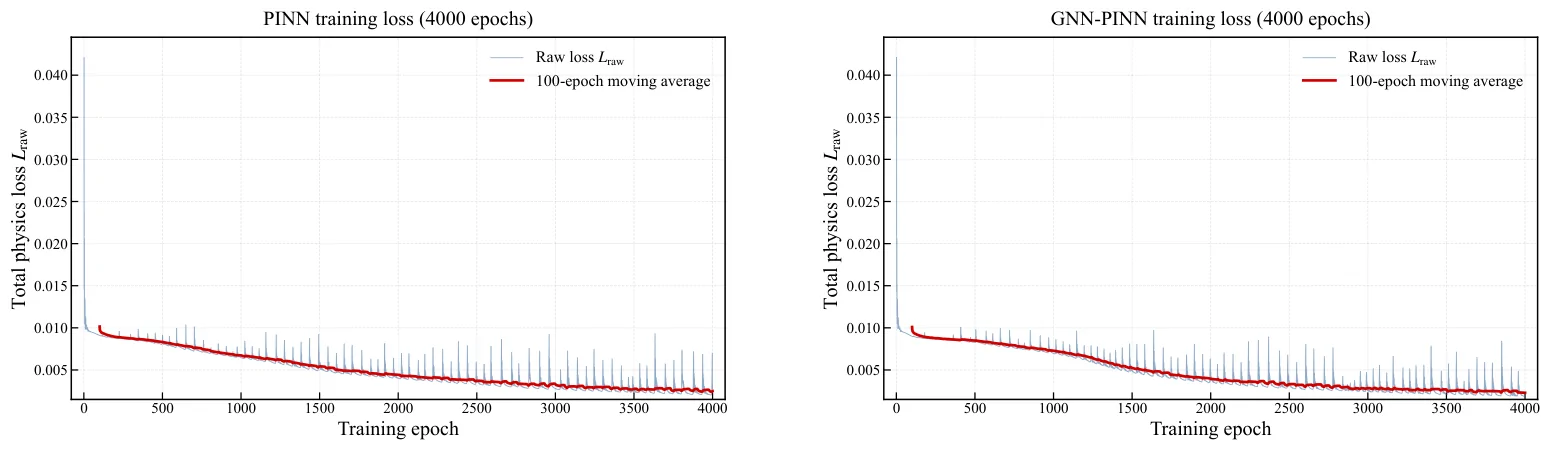
Loss curves over 4000 training epochs: baseline PINN (**left**) and GNN-PINN (**right**). The curve endpoints are checked against the sum of the terminal loss components, $L_{\mathrm{raw}}$.

To examine whether the advantage of the graph-enhancement branch persists with longer training, both models are trained for 4000 epochs. Figure 6 shows that both loss curves retain an overall downward trend beyond 2000 epochs, together with local peaks associated with sampling and optimization updates.

At 4000 epochs, the full space–time MSE is $1.235\times 10^{-3}$ for the baseline PINN and $8.551\times 10^{-4}$ for GNN-PINN, corresponding to a 30.8% reduction. The associated root mean squared error (RMSE) decreases from $3.515\times 10^{-2}$ to $2.924\times 10^{-2}$, a reduction of 16.8%. The terminal $L_{\mathrm{raw}}$ decreases from $2.806\times 10^{-3}$ to $2.521\times 10^{-3}$, a reduction of 10.2%. Extending training from 2000 to 4000 epochs reduces MSE by 32.7% for the baseline PINN and by 47.0% for GNN-PINN, indicating that the relative accuracy gain of graph enhancement increases with the training budget considered here. The loss components show that, at 4000 epochs, the initial and boundary losses of GNN-PINN are 24.9% and 5.0% lower than those of the baseline PINN, respectively.

### 4.5. Qualitative Comparison of Wavefield Reconstruction at 4000 Epochs

**Figure 7:**
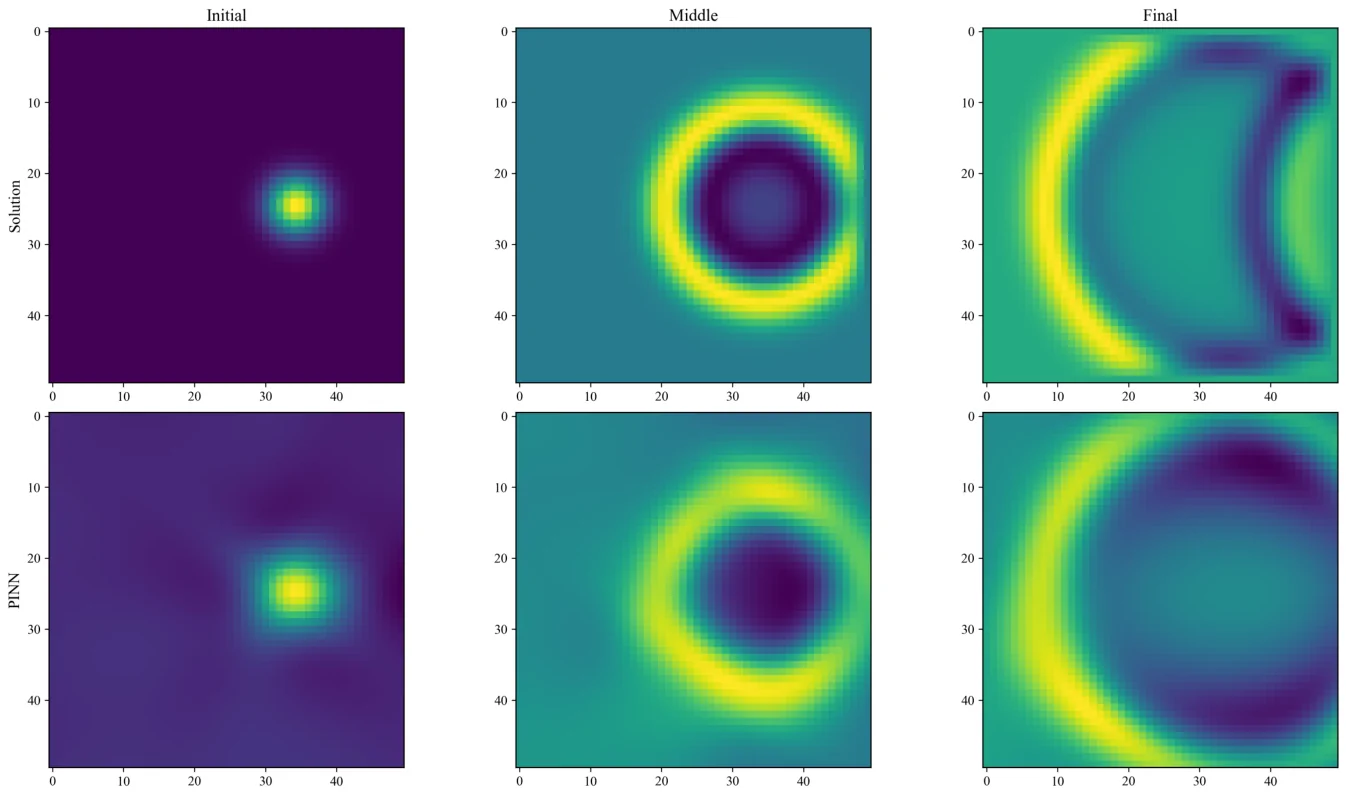
Two-dimensional wavefield predictions of the baseline PINN after 4000 epochs. (**Top** row): numerical reference solution. (**Bottom** row): PINN prediction. Columns show the initial, intermediate, and final times.

**Figure 8:**
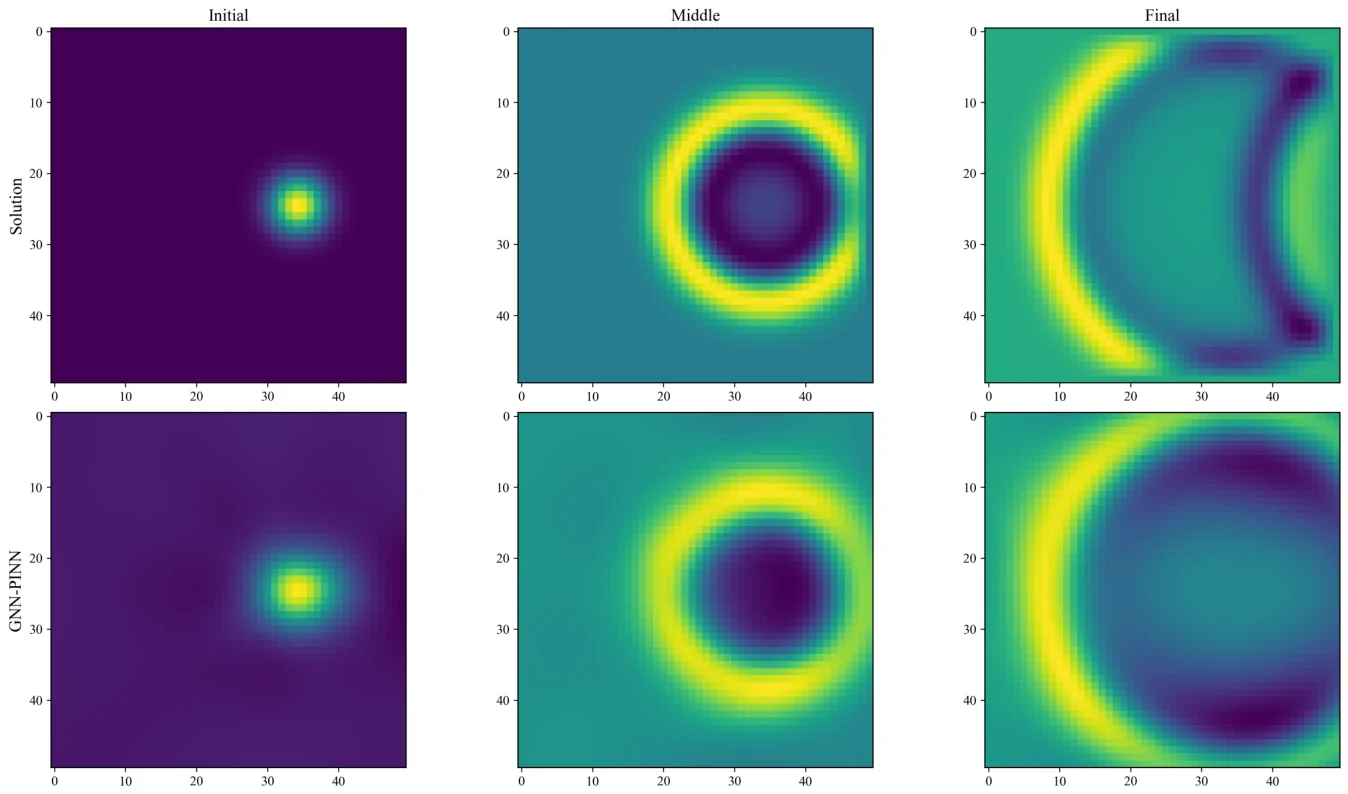
Two-dimensional wavefield predictions of GNN-PINN after 4000 epochs. (**Top** row): numerical reference solution. (**Bottom** row): GNN-PINN prediction. Columns show the initial, intermediate, and final times.

Figure 7 and Figure 8 show the baseline PINN and GNN-PINN wavefield predictions after 4000 epochs. Compared with the 2000-epoch results, both models exhibit clearer ring-shaped structures at the intermediate time and clearer large-scale reflections at the final time.

At 4000 epochs, the baseline PINN represents the intermediate ring-shaped wavefront more completely, but its initial pulse remains broadened, and its final upper and lower negative-amplitude regions and right-boundary details remain overly smooth. GNN-PINN produces a clearer ring-shaped wavefront, left-side positive-amplitude arc, and vertically symmetric negative-amplitude regions, consistent with its lowest full space–time MSE of $8.551\times 10^{-4}$. Across the four experiments, longer training benefits both models, but the GNN-PINN error decreases more rapidly.

### 4.6. Unified Comparison of Wavefields and Absolute Errors

**Figure 9:**
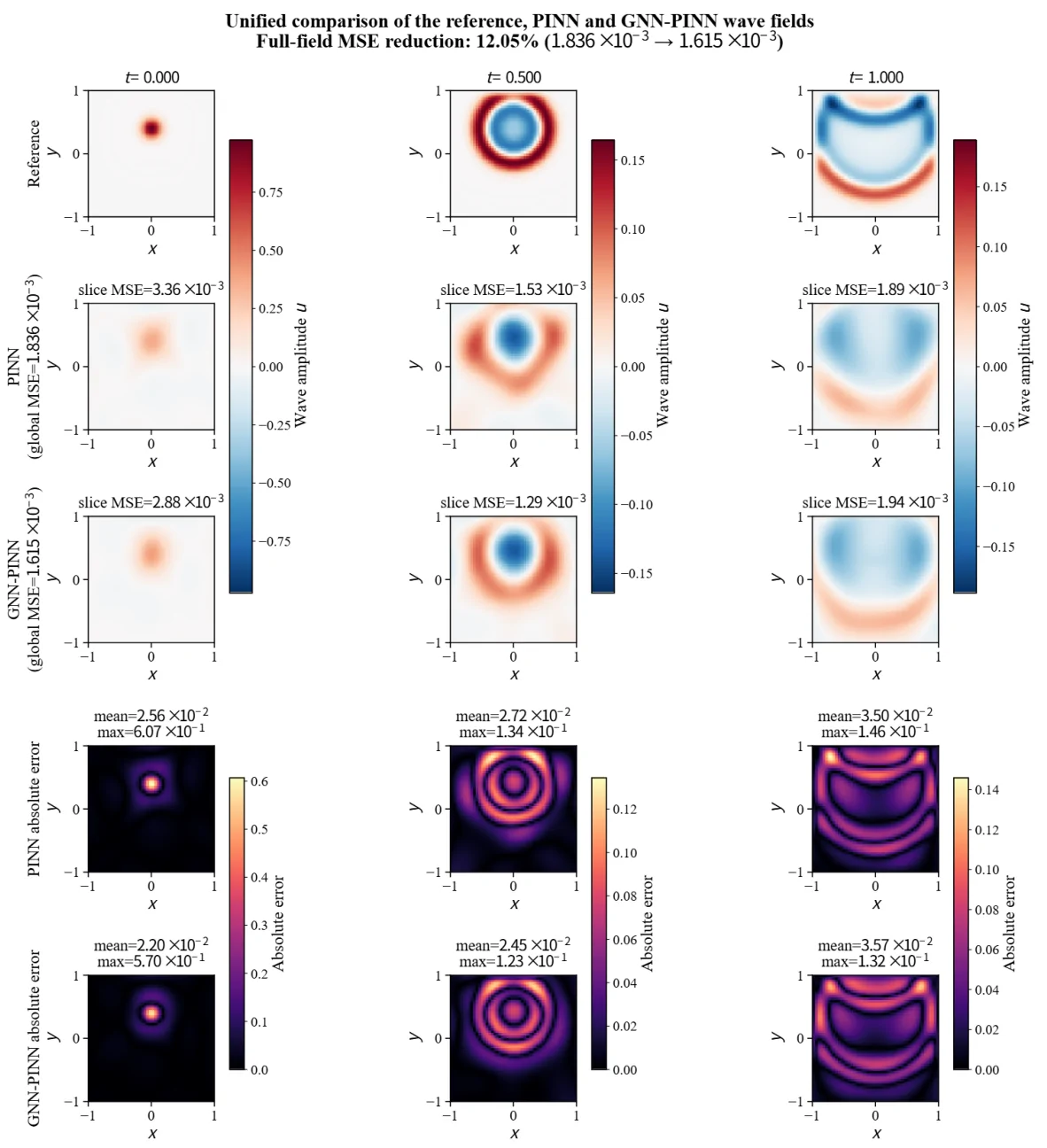
Unified comparison of the reference wavefield, baseline PINN, GNN-PINN, and absolute errors after 2000 training epochs.

**Figure 10:**
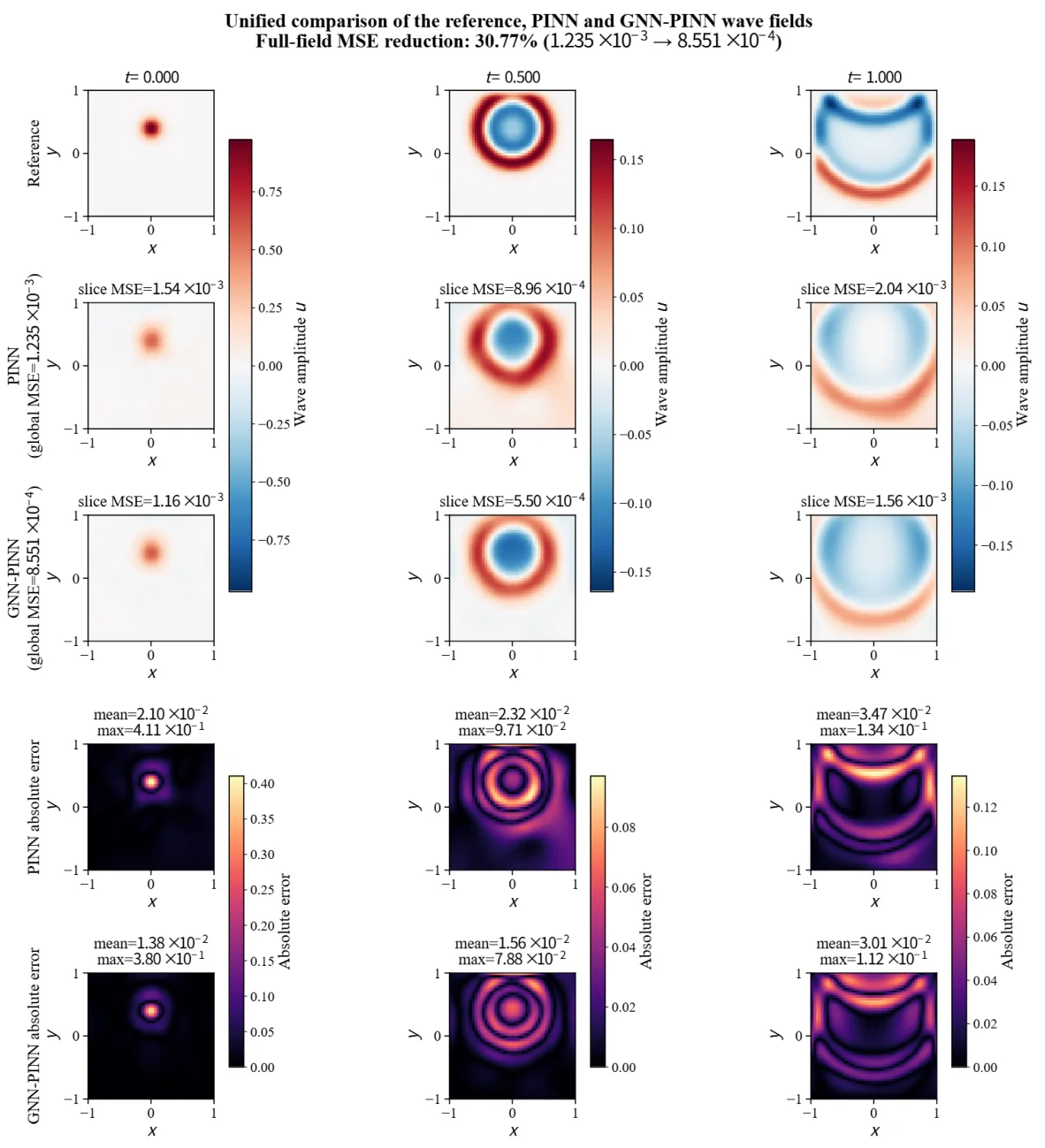
Unified comparison of the reference wavefield, baseline PINN, GNN-PINN, and absolute errors after 4000 training epochs.

To avoid visual bias from independent color scales, Figure 9 and Figure 10 jointly present the numerical reference, baseline PINN, GNN-PINN, and absolute errors of both models at the same representative times and over the same coordinate ranges, using a common wavefield color scale within each column.

The unified comparisons show that GNN-PINN reduces the full space–time MSE by 12.05% and 30.77% relative to the baseline PINN at 2000 and 4000 epochs, respectively. The absolute-error maps further show that errors are concentrated near the initial pulse, propagating wavefronts, and boundary reflections. Graph enhancement reduces error intensity in most representative regions, although deviations persist locally in regions with large gradients.

### 4.7. One-Dimensional Wavefield Profile Comparison

**Figure 11:**
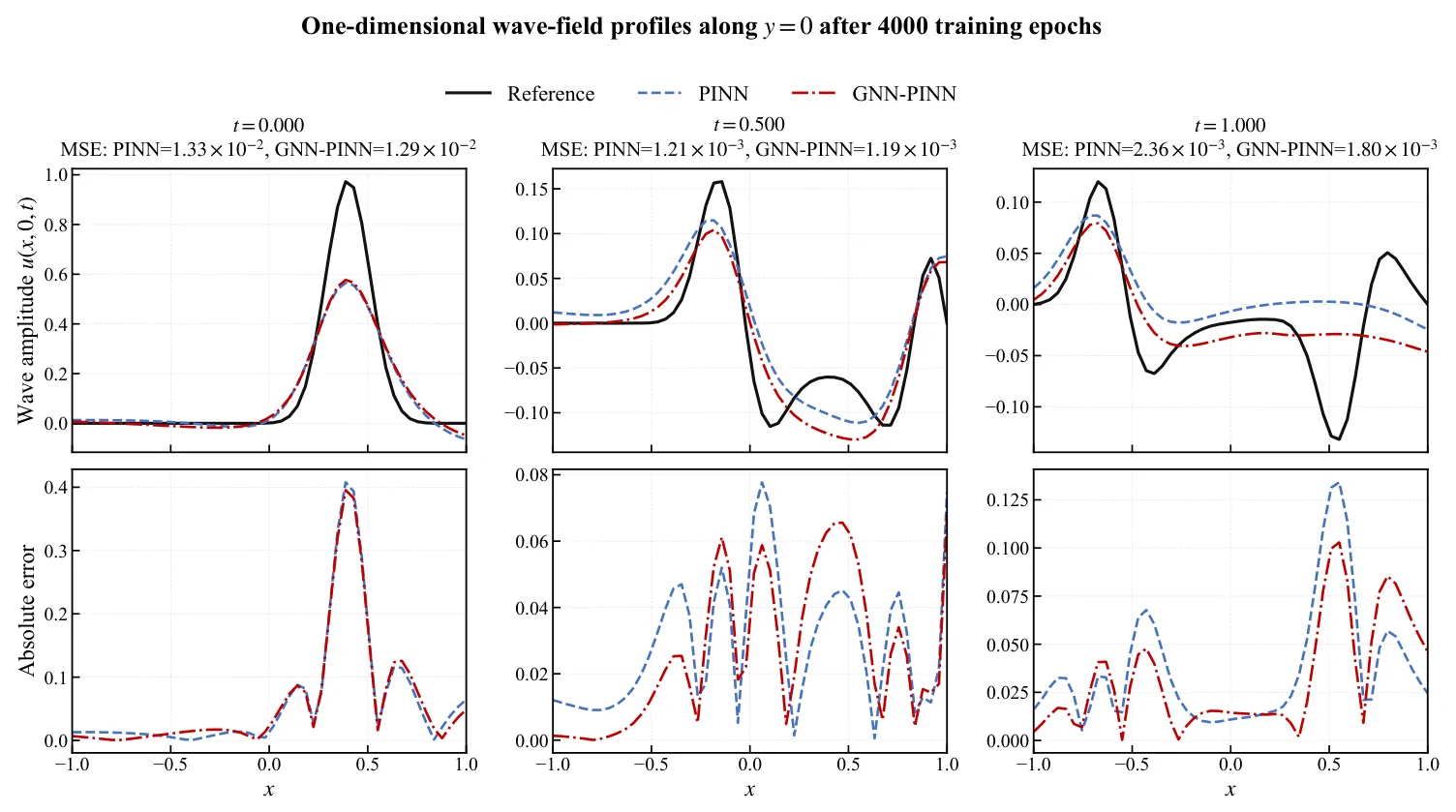
One-dimensional wavefield profiles along the horizontal centerline y=0 after 4000 training epochs. (**Top** row): reference solution, baseline PINN, and GNN-PINN at t=0, 0.5, and 1.0. (**Bottom** row): corresponding absolute errors of the two models.

To provide a direct profile-level assessment, Figure 11 compares the reference solution, baseline PINN, and GNN-PINN along the horizontal centerline y=0 at t=0, 0.5, and 1.0 after 4000 training epochs. The centerline MSE reductions of GNN-PINN relative to the baseline PINN are 3.60%, 1.80%, and 23.70%, respectively. The largest improvement occurs at t=1.0, where boundary reflection produces a more complex wavefield. The reduced error peaks in the profile plots agree with the full space–time MSE results and further demonstrate that the graph branch improves the reconstruction of propagating and reflected wave structures while retaining the continuous representation of the PINN backbone.

### 4.8. Computational Cost and Training-Time Analysis

To provide a more balanced comparison of computational cost, an additional PINN with seven hidden layers is used as a deeper reference model for this timing comparison. Its network depth approximately corresponds to the five-layer PINN backbone and two-layer graph encoder in GNN-PINN. This configuration makes the two models more comparable in terms of overall network depth and training cost. Table 5 reports the training times at four training budgets.

After 4000 training epochs, the two models require almost the same training time: 381.929 s for the seven-hidden-layer PINN and 381.306 s for GNN-PINN. These results provide a basis for subsequent performance comparisons at similar computational cost.

The 2000- and 4000-epoch comparisons were used to assess the effect of graph enhancement under shorter training budgets. At the same number of epochs, GNN-PINN reduced the full space–time MSE by approximately 12% and 30.8%, respectively, compared with the standard PINN, indicating that spatial graph information improves prediction accuracy even with limited training. At 4000 epochs, the GNN-PINN MSE had reached $8.551\times 10^{-4}$, below $10^{-3}$ for the present test case. To examine the benefit of further training, we evaluated GNN-PINN at 8000 epochs, when its MSE decreased to $4.241\times 10^{-4}$. Thus, additional training continued to reduce prediction error. The measured training time at 8000 epochs was approximately twice that at 4000 epochs. Whether the longer training budget is warranted therefore depends on the accuracy requirements and computational cost of the intended application.

In the present study, the initial displacement is a localized Gaussian pulse, the initial velocity is zero, and homogeneous Dirichlet boundary conditions are imposed. Previous research has shown that the way initial and boundary conditions are imposed affects the accuracy of PINNs for wave equations [12]. Another physics-informed graph method has been applied to a wave problem on an irregular domain with free boundary conditions [32]. These studies motivate evaluating the proposed method under other conditions, but do not establish that its advantage over a standard PINN would persist. Such conditions were not examined here. Future work will compare the two methods under matched sampling and training settings.

## 5. Conclusions and Future Work

This study proposes a GNN-enhanced PINN framework based on constrained graph-to-point coupling to integrate continuous field representations with discrete spatial neighborhood information when solving the two-dimensional wave equation.

Under consistent sampling, optimization, and evaluation settings, the complete coupled framework reduces the full space–time mean squared error relative to the baseline PINN by 12.1% and 30.8% at 2000 and 4000 training epochs, respectively.

The present results also provide a methodological basis for examining the framework in more complex materials. In the homogeneous benchmark, the complete coupling improves both full space–time accuracy and the reconstruction of the expanding wavefront and later boundary-reflection structures. Thus, its benefit is visible in spatially organized propagation features as well as in the aggregate error. This observation is consistent with the roles of the proposed components: the eight-neighbor graph extracts local context from the initial spatial field, differentiable bilinear mapping supplies that context to collocation points beyond the graph nodes, and the continuous PINN predicts the wavefield and evaluates its PDE residual without graph-based time stepping. The analytical envelope and gated residual fusion allow the graph correction to enter the solution without altering the PINN branch’s initial and boundary values. In heterogeneous media, material contrasts and interfaces generate localized changes in propagation and additional transmitted and reflected waves. Encoding material properties and interface relationships in the graph could therefore provide relevant spatial context to the continuous solution in these more demanding regions. Previous studies of layered and complex wave media [11,16], bimaterial interfaces [14], and graph-based material-field representations [25] support investigating this extension. Future work will add material and interface descriptors to the graph, adopt the appropriate variable-coefficient or elastodynamic equations and interface conditions, and examine whether graph aggregation and graph–point interpolation preserve the behavior on either side of an interface. The framework will then be tested progressively in graded, layered, and inclusion-containing media against independent numerical reference solutions and a standard PINN under matched training settings. Global and interface-localized errors, transmitted and reflected wavefronts, and computational cost will be compared to determine whether the present gains persist and to assess potential applications in ultrasonic inspection and material characterization.

## Figures and Tables

**Table 1:** Principal notation and its role in the proposed formulation.

Symbol	Definition	Role
$u,u_{\mathrm{PINN}},\hat{u}$	True, base, and fused wavefields	Field representation and evaluation
Ω,T,c	Spatial domain, terminal time, and wave speed	Wave initial boundary value problem
$G,G,N_{g}$	Spatial graph, nodes per direction, and total nodes	Total node count $N_{g}=G^{2}$
$u_{0},u_{0}^{h}$	Analytical and discrete initial displacements	Graph features and initial-condition loss
$H_{G},g\left(x,y\right)$	Node embedding matrix and continuous graph context	Linking the graph and continuous collocation points
$\delta u_{\omega },\psi ,\alpha$	Residual correction, constraint envelope, and global gate	Constrained residual fusion
$L_{\mathrm{IC}},L_{\mathrm{BC}},L_{\mathrm{PDE}}$	Initial, boundary, and PDE residual losses	Physics-informed training objective

**Table 2:** Components and parameters of the final GNN-PINN model.

Component	Input	Structure	Parameters/Output
Continuous PINN	(x,y,t), 3 dimensions	5×100, tanh	40,901; $u_{\mathrm{PINN}}$
Fixed spatial graph	2500 nodes	Eight neighbors and self-loops	Nontrainable buffers
GCN encoder	3-dimensional node features	3→32→32, LN and tanh	1312; $H_{G}$
Bilinear interface	$H_{G}$ and (x,y)	Four-node weighting	No parameters; g(x,y)
Correction network	35 dimensions	100→100→1, tanh	13,801; $\delta u_{\omega }$
Gate and fusion	$u_{\mathrm{PINN}},\delta u_{\omega }$	σ(γ)ψ and residual addition	1; $\hat{u}$ (56,015 total)

**Table 3:** Main settings for the baseline PINN and GNN-PINN experiments.

Setting	PINN	GNN-PINN	Implementation Note
Training epochs	2000/4000	2000/4000	Two iteration budgets
PINN backbone	3→5×100→1	3→5×100→1	Same backbone
Initial points $N_{i}$	2000	2000	Same initial-point count
Boundary points per edge	1000	1000	Count per boundary
Interior points $N_{f}$	20,000	20,000	Same collocation-point count
Initial learning rate	$1.5\times 10^{-3}$	$1.5\times 10^{-3}$	Same initial learning rate
Adam parameters	$\beta_{1}=0.9$, $\beta_{2}=0.99$	$\beta_{1}=0.9$, $\beta_{2}=0.99$	Same Adam parameters
Learning-rate scheduler	ReduceLROnPlateau	ReduceLROnPlateau	Same scheduling rule
Interior-point refresh	25% every 100 epochs	25% every 100 epochs	Same dynamic resampling
Gradient clipping	Norm limit of 10	Norm limit of 10	Same gradient clipping

**Table 4:** Terminal loss and full space–time MSE at different training budgets.

Model/Epochs	$L_{\mathrm{raw}}$	Full Space–Time MSE
PINN/2000	$4.040\times 10^{-3}$	$1.836\times 10^{-3}$
GNN-PINN/2000	$3.697\times 10^{-3}$	$1.615\times 10^{-3}$
PINN/4000	$2.806\times 10^{-3}$	$1.235\times 10^{-3}$
GNN-PINN/4000	$2.521\times 10^{-3}$	$8.551\times 10^{-4}$

**Table 5:** Training-time comparison between the seven-hidden-layer PINN reference model and GNN-PINN. All times are reported in seconds.

Model	1000 Epochs	2000 Epochs	3000 Epochs	4000 Epochs
PINN (7 hidden layers)	88.954	184.373	285.943	381.929
GNN-PINN	100.859	190.145	283.062	381.306

## Data Availability

Some or all data, models, or code that support the findings of this study are available from the corresponding author upon reasonable request.
